# Large-Scale Assessment of the Effect of Popularity on the Reliability of Research

**DOI:** 10.1371/journal.pone.0005996

**Published:** 2009-06-24

**Authors:** Thomas Pfeiffer, Robert Hoffmann

**Affiliations:** 1 Program for Evolutionary Dynamic, Harvard University, Cambridge, Massachusetts, United States of America; 2 Computer Science and Artificial Intelligence Laboratory, Massachusetts Institute of Technology (MIT), Cambridge, Massachusetts, United States of America; University of Exeter, United Kingdom

## Abstract

Based on theoretical reasoning it has been suggested that the reliability of findings published in the scientific literature decreases with the popularity of a research field. Here we provide empirical support for this prediction. We evaluate published statements on protein interactions with data from high-throughput experiments. We find evidence for two distinctive effects. First, with increasing popularity of the interaction partners, individual statements in the literature become more erroneous. Second, the overall evidence on an interaction becomes increasingly distorted by multiple independent testing. We therefore argue that for increasing the reliability of research it is essential to assess the negative effects of popularity and develop approaches to diminish these effects.

## Introduction

Even if conducted at best possible practice, scientific research is never entirely free of errors. When testing scientific hypotheses, statistical errors inevitably lead to false findings. Results from scientific studies may occasionally support a hypothesis that is actually not true, or may fail to provide evidence for a true hypothesis. The probability at which a hypothesis is true after a certain result has been obtained (posterior probability) depends on the probabilities at which these two types of errors arise. Therefore, error probabilities, such as p-values, traditionally play a predominant role for evaluating and publishing research findings. The posterior probability of a hypothesis, however, also depends on its prior probability. Positive findings on unlikely hypotheses are more likely false positives than positive findings on likely hypotheses. Thus, not only high error rates, but also low priors of the tested hypotheses increase the frequency of false findings in the scientific literature [1], [2].

In this context, a high popularity of research topics has been argued to have a detrimental effect on the reliability of published research findings [2]. Two distinctive mechanisms have been suggested: First, in highly competitive fields there might be stronger incentives to “manufacture” positive results by, for example, modifying data or statistical tests until formal statistical significance is obtained [2]. This leads to inflated error rates for individual findings: actual error probabilities are larger than those given in the publications. We refer to this mechanism as “inflated error effect”. The second effect results from multiple independent testing of the same hypotheses by competing research groups. The more often a hypothesis is tested, the more likely a positive result is obtained and published even if the hypothesis is false. Multiple independent testing increases the fraction of false hypotheses among those hypotheses that are supported by at least one positive result. Thereby it distorts the overall picture of evidence. We refer to this mechanism as “multiple testing effect”. Putting it simple, this effect means that in hot research fields one can expect to find some positive finding for almost any claim, while this is not the case in research fields with little competition [1], [2].

The potential presence of these two effects has raised concerns about the reliability of published findings in those research fields that are characterized by error-prone tests, low priors of tested hypotheses and considerable competition. It is therefore important to analyze empirical data to quantify how strong the predicted effects actually influence scientific research.

Here, we assess a large set of published statements on protein interactions in yeast (*S. cerevisiae*) with data from recent high-throughput experiments. Published statements on protein interactions are obtained from data stored in publication databases. We analyze whether there is a relation between the reliability of published interactions and the popularity of the interaction partners. In our analysis, individual literature statements on interactions are treated as results from individual studies, while the presence of an interaction is considered as a testable hypothesis. Several statements in the literature may, for example, indicate that there is an interaction between protein A and B. Whether this interaction really exists is a hypothesis that might be either true or false.

### Datasets

A considerable part of publications in molecular biology and related research fields investigate protein interactions because they are important to understanding the relation between the genotype of an organism, its environment, and its phenotype. Typically, these publications focus on simple systems consisting of a few proteins that together fulfill a specific function, and report interactions as inferred from small-scale experiments. Published statements from such small-scale experiments can be obtained with text mining approaches, or can be inferred by experts from scientific publications. Our study is based on both text mined and expert curated data. We use the text mining system iHOP to identify published interactions between proteins and genes in titles and abstracts from the PubMed database. A detailed description of iHOP has been given earlier [3], [4]. For yeast we obtain more than 60,000 published statements on more than 30,000 unique interactions.

Expert-curated data from IntAct [5] and DIP [6], [7] are used as an additional source for published statements on protein interactions. DIP and IntAct are leading databases for protein interactions that contain expert-curated interactions from the literature and results from high-throughput experiments. To exclude data from high-throughput experiments and only include expert-curated interactions from small-scale experiments we only use those DIP and IntAct interactions that come from experiments with less than 100 interactions per publication. The resulting expert-curated set contains more than 6,000 statements on more than 4,000 interactions.

These published statements on protein interactions can be evaluated using data from recent high-throughput techniques. We use datasets from yeast-two-hybrid experiments (Y2H [8], [9]; 2,981 interactions), high-throughput mass spectroscopy (HMS [10]; 16,896 interactions), tandem affinity purification (TAP [11]; 25,616 interactions), and a recently published approach that combines mass-spectroscopy and affinity purification (COM [12]; 67,284 interactions). These high-throughput techniques have been shown to be highly informative, although they are not free of errors and biases either [13]. In fact, error rates are likely much higher for high-throughput experiments than for well-performed small-scale experiments. High-throughput experiments, however, test nearly all interactions simultaneously, and their error-rates do not depend on the popularity of individual proteins. Therefore they are not influenced by the “multiple testing effect” or the “inflated error effect”, and are highly suitable for detecting both effects. Data from high-throughput experiments are obtained from the IntAct database [5]. For protein complexes with more than two interaction partners we use all pair-wise interactions for comparison of different datasets, as has been done in previous studies [13].

The popularity of a protein, or the corresponding gene, can be estimated by the frequency at which it appears in the literature. Previous studies show that there are large differences in the frequencies of proteins in the literature [14], [15]. This makes data on protein interaction ideal to study popularity effects in published research.

## Results

About 17% of the individual statements from the unfiltered iHOP dataset are confirmed by at least one of the high-throughput techniques. This appears to be relatively small. However, it has to be considered that some types of interactions, such as protein-promoter interactions, genetic linkage or epistatis, are frequently described in scientific publications but cannot be obtained with the current high-throughput experiments. Moreover, the overlap is much higher than random (<1%), which suggests that high-throughput techniques are sufficiently informative to generate a relative reliability measure for published interactions. For the expert-curated data from DIP and IntAct which specifically target protein interactions, confirmation is about 46%. This is higher than for the iHOP set, but on the other hand iHOP contains about ten times more published statements than IntAct and DIP.

**Figure 1:**
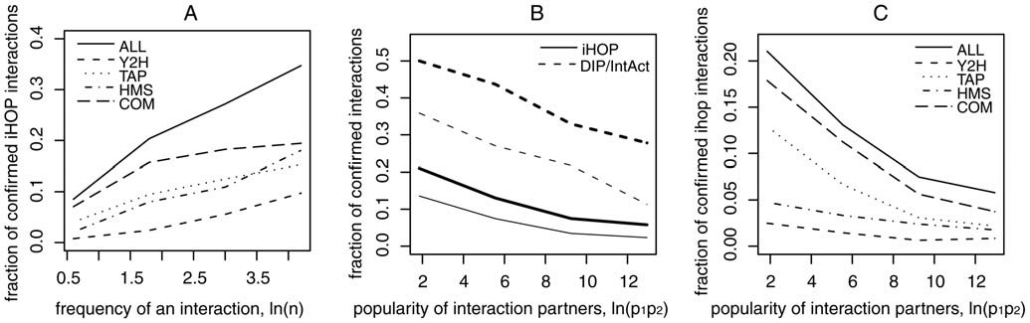
Relation between the frequency of interactions in the literature and the fraction of interactions confirmed by high-throughput techniques. Interactions that are described frequently in the literature tend to be confirmed more frequently. Because one would expect that interactions are more reliable if they are repeated often in the literature, this finding indicates that a comparison with high-throughput experiments is suitable for evaluating published interactions. B. Relation between the frequencies of the interaction partners in the literature and the fraction of confirmed interactions. Published interactions are obtained from text mining approaches (iHOP) and from expert-curated data (DIP and IntAct). For both datasets, the probability that an interaction is confirmed by a high-throughput experiment decreases with increasing popularity of the interaction partners. Bold lines code for the fraction of published interactions confirmed by at least one experimental technique, thin lines code for confirmation by at least two techniques. Thus, while interactions that are frequent in the literature tend to be more reliable (Fig. 1A), interactions of proteins that are frequent in the literature tend to be less reliable. C. Popularity of interaction partners vs. fraction of confirmed iHOP interactions for different experimental techniques. The negative correlation between the probability of experimental confirmation and popularity of the interaction partners is present for all different experimental techniques.

While for most interactions there is only one statement in PubMed, some interactions appear several times. For yeast, the most frequent interaction described in the literature is between actin (ACT1) and myosin (MYO1), and is stated about 100 times. Fig. 1A shows that interactions that are described often in the literature tend to be confirmed more frequently by high-throughput experiments. Interactions that appear less than three times are confirmed at a probability of 8%, while interactions that are repeated more than 50 times are confirmed at a probability of up to 40%. This result illustrates that the overlap with high-throughput data is indeed a good indicator for the reliability of published interactions, since interactions that are repeated many times in the literature can be expected to be more reliable. It also shows that even for interactions that are very frequent in the literature, the majority is still not confirmed by high-throughput experiments which might be because high-throughput experiments do not capture all types of interactions described in the literature (see above).

### Popularity vs. reliability of protein interactions

Based on these datasets we investigate how the popularity of a protein relates to the reliability of its interactions described in the literature. To study the “inflated error effect”, we analyze the relation between the reliability of *individual* literature statements and popularity. We assume that the probability *w_ij_* for a literature statement to be confirmed by at least one of the high-throughput experiments depends on the frequency *P_i_* and *P_j_* of the interaction partners in the literature. Using a logistic regression on the model *ln(w_ij_/(1−w_ij_)) = a+b ln(P_i_P_j_)*, we observe a highly significant negative correlation between confirmation probability *w_ij_* and log-transformed popularities of the interaction partners (a = −1.41±0.04; b = −0.020±0.005, p = 4.8*10^−5^; N = 62,864 statements). A similar result is obtained when using individual statements from DIP and Intact (a = 0.39±0.09, b = −0.08±0.01, p = 4*10^−11^; N = 6,494 statements). Thus we conclude that individual literature statements on interactions are less reliable for more popular genes.

To quantify how popularity influences reliability through the “multiple testing effect”, we use a similar approach. We assume that the probability *w_ij_* that an interaction that appears *at least once* in the literature is confirmed by at least one high-throughput experiments depends on the frequency *P_i_* and *P_j_* of the interaction partners in the literature. We observe a negative correlation between confirmation probability *w_ij_* and log-transformed popularities of the interaction partners (a = −0.69±0.07, p<2.2*10^−16^; b = −0.20±0.01, p<2.2*10^−16^; N = 30,446 interactions). Thus, there is very strong evidence for the “multiple testing effect”. Interactions of highly popular proteins tend to be confirmed by high-throughput experiments at much lower frequency than interactions of un-popular proteins (see Fig. 1B). This relation is also present when expert-curated interactions from DIP and IntAct are evaluated instead of iHOP interactions (Fig. 1B), and when iHOP interactions are assessed by different experimental techniques individually (Fig. 1C). Given that different experimental techniques are influenced by different technical biases, and expert-curated interactions have different methodological problems than text mining approaches, it is unlikely that the observed relation between reliability and popularity is driven by confounding factors.

## Discussion

The reliability of research is typically investigated by meta-analyses that synthesize data from published studies on the same set of questions. Such meta-studies depend on the manual assessment of statistical details from each study, which limits this approach to a few hundred studies. In contrast to traditional meta-studies, our approach is based on massive data from publication databases and data mining. Although it cannot incorporate statistical details from individual publications, it allows us to examine a very large data set with ten thousands of statements from the scientific literature. We believe that such an approach is an important and innovative complement to more traditional methods.

Our approach allows us to provide evidence for two effects of a high popularity on the reliability of research. First, we find that individual results on yeast protein interactions as published in the literature become less reliable with increasing popularity of the interacting proteins (inflated error effect). This is disquieting because one plausible possibility to explain this effect is “significance seeking”. Second, we find evidence for a negative effect of a high popularity due to multiple independent testing. Interactions that are obtained at least once in the literature are less likely confirmed by high-throughput experiments if the interaction partners are more popular. The second effect is about 10 times larger than the first one.

Based on our approach, it is difficult to distinguish between false positives and true positives of little relevance. It is likely that for popular genes with many interaction partners, not all interactions are of equal relevance. Some interactions may, for example, be only relevant under specific experimental conditions and therefore do not show up in high-throughput experiments. Thus, part of the negative relation between popularity and reliability might be driven by a negative relation between popularity and relevance. Nevertheless, the observed decrease of about 50% in the confirmation probability for interactions of popular proteins indicates that the effects of competition and multiple independent testing on the reliability of research cannot be neglected. When interpreting results, the popularity of a research topic has to be taken into account. This will require increased efforts to determine how much research is performed on which hypotheses, and how this information can be incorporated into the synthesis of research.

Counteracting the formation of scientific “hypes” might diminish some of the problems that result from a high popularity of research topics. For instance, some of the funding available in scientific research could be specifically directed towards promising projects on topics of currently low popularity. The current dynamics of scientific research, however, seems to favor a certain degree of herding [15], [16]. Therefore, mechanisms, which translate a high popularity into a high reliability, must be facilitated.

More emphasize could be given to either pre or post publication evaluation. The current model of pre publication peer review, however, can hardly be intensified. Post publication evaluation, on the other hand, shows a promising potential to increase the reliability of scientific knowledge. A recent study, for example, indicates that flaws in scientific publications are more likely detected post publication if they appear in high impact journals [17].

The interactive web or Web 2.0 provides the means to formalize and facilitate post publication evaluation involving larger parts of the scientific community. In our opinion, collaborative systems such as wikis are particularly noteworthy applications [18]–[20]. Because wikis for scientific content are relatively novel, data are not yet available to examine their performance in improving the reliability of scientific knowledge. However, in domains outside of science, wikis have proven their capabilities to reliably disseminate knowledge [18]. In the context of post publication evaluation, wikis with unambiguous authorship attribution [19] could integrate community review activity directly into improved versions of publications instead of relying on journals to publish errata. In addition, the low effort required for publishing in wikis might help to make negative findings more visible and thereby reduce the impact of false positives that result, for example, from multiple independent testing.
